# Key features of adolescent inpatient units and development of a checklist to improve consistency in reporting of settings

**DOI:** 10.1111/jpm.12856

**Published:** 2022-07-18

**Authors:** Claire Hayes, Magenta Simmons, Victoria Palmer, Bridget Hamilton, Christine Simons, Malcolm Hopwood

**Affiliations:** ^1^ Nursing and Midwifery Monash University Melbourne Vic. Australia; ^2^ The National Centre of Excellence in Youth Mental Health The University of Melbourne Melbourne Vic. Australia; ^3^ Orygen Youth Health The University of Melbourne Melbourne Vic. Australia; ^4^ Department of General Practice The University of Melbourne Melbourne Vic. Australia; ^5^ Centre for Psychiatric Nursing The University of Melbourne Melbourne Vic. Australia; ^6^ The Albert Road Clinic Melbourne Vic. Australia; ^7^ Department of Psychiatry The University of Melbourne Melbourne Vic. Australia; ^8^ Department of Psychiatry, The Albert Road Clinic The University of Melbourne Melbourne Vic. Australia

**Keywords:** Adolescent, Child, Inpatient Units, Mental Health, Model of Care

## Abstract

**What is known on the subject?:**

- Little is known about adolescent inpatient units, key features which define them, and how these essential services operate and deliver care.

**What the paper adds to existing knowledge?:**

- Adolescent inpatient unit studies are limited in their descriptions of settings in terms of how they operate and key features.
- The proposed preliminary checklist is a practical tool to assist clinicians, policy makers, and researchers when reporting to ensure comprehensive descriptions of adolescent inpatient settings.

**What are the implications for practice?:**

- This could be used to inform service design processes for inpatient and other mental health service models which is of critical importance in the context of reforms and implementation of these in Australia currently.
- Greater attention to operational models, services, and philosophies of practice will improve reporting and allow for the advancement of knowledge, comparison of study results, and a clearer direction for mental health nursing clinicians and researchers.

**Abstract:**

**Introduction**

Adolescent inpatient units care for vulnerable population groups; however, little is known about how these essential services operate and deliver care.

**Aims**

To examine the descriptions of adolescent mental health inpatient units in Australian and international research publications and to identify key features which were used to define them. A secondary aim was to develop a checklist to improve consistency when reporting on the operations and services delivered within adolescent mental health inpatient units (both public and private).

**Methods**

Five electronic databases (CINAHL, MEDLINE, ERIC, EMBASE, and PsycINFO) were systematically searched. We included studies that provided descriptions of operations and services within adolescent inpatient units where participants had a mean age between 12 and 25. Narrative synthesis was used to explore the similarities and differences between descriptions of settings.

**Results**

Twenty‐eight studies were identified, which varied in their descriptions of adolescent inpatient units, providing inconsistent information to inform best practice.

**Discussion**

Studies lack consistency and comprehensive detail when describing the operational models within inpatient units, making interpretation challenging. Consequently, a preliminary checklist is proposed to improve reporting of adolescent inpatient units.

## 1 INTRODUCTION

Since March 2020, healthcare systems and settings (particularly hospital‐based services) have faced additional challenges with the outbreak of the novel coronavirus (COVID‐19) (Delaney, 2021). The COVID‐19 global pandemic had caused nurses to be challenged beyond their own limits, adjusting to a “new normal” (Delaney, 2021; Maben & Bridges, 2020, p. 2742). Evidence from the United Kingdom (UK) and United States (US) report that psychological distress symptoms have increased in the community during the pandemic, with those aged 13 to 26 years exhibiting the greatest deterioration (McGinty et al., 2020; O'Reilly et al., 2020; Pierce et al., 2020; Wisconsin Office of Children's Health, 2021). Mental health services transitioned in a matter of days from face‐to‐face care to telehealth and virtual modes of delivery (Maben & Bridges, 2020). The effects of COVID‐19 are likely to have long‐lasting impacts for physical and mental healthcare systems (McBride et al., 2021).

The need for transparency in reporting of healthcare settings pre‐dated the pandemic and is more important than ever in assisting healthcare systems navigate through the coming years and additional challenges related to the lasting consequences of COVID‐19.

Although community care is prioritized over inpatient treatment for adolescents, the acuity and severity of clinical symptoms may necessitate this level of care (Carlson & Elvins, 2021). Adolescent inpatient units are beneficial when they provide specialist care that other components of the system of care cannot (Hazell, 2021). Inpatient admissions are often required when all other treatment options have been attempted (Carlson & Elvins, 2021) or there is no alternative available to meet the severity of a person's needs. Admissions typically occur for the purpose of (a) detailed observation to facilitate diagnosis, (b) to initiate supervised treatment, and/or (c) acute containment of risk (Hazell, 2021; Perkes et al., 2019). While there has been a proliferation of recent articles focusing on specific therapies such as Dialectical Behaviour Therapy (DBT) or Cognitive Behaviour Therapy (CBT) that may be used in these settings, research that describes operations and models of care within child and adolescent inpatient units remains limited (Delaney, 2019; Hayes et al., 2020a, 2020b; Hayes, Palmer, Hamilton, et al., 2019; Hayes, Palmer, Simmons, et al., 2019; Hayes, Simmons, Palmer, et al., 2019; Saito et al., 2020).

Existing research tends to address characteristics influencing admissions, such as length of stay or therapeutic outcomes following an admission (Benarous et al., 2021). Less is known about adolescent inpatient units, such as their key features and how and why these specialist programs address major mental health issues (in terms of how they operate and function) (Delaney, 2019; Hayes et al., 2018; Mojtabai et al., 2016). Key features can include the mean age of adolescents admitted, voluntary or involuntary units, and group therapy sizes for example. Furthermore, less is known about the inpatient unit philosophies of practice, services, and therapeutic offerings such as types of group therapies and discharge support services including links to community supports. There are some communities where general adolescent inpatient units are unavailable or inaccessible. Consequently, adolescents are often admitted to adult services or receive no service (McRae et al., 2021).

Globally, countries will vary in terms of adolescent age ranges, with many overlapping ranges from birth to adulthood (Clark et al., 2015). In Australia, “there are no universally accepted paediatric definitions within health” (Clark et al., 2015, p.1011). However, from a mental health perspective, adolescent services in Australia typically admit those from ages 12 to 25 (Garner et al., 2008; Hayes et al., 2018, 2020b; Hayes, Palmer, Hamilton, et al., 2019; Hense et al., 2018). To our knowledge, there are little or no inpatient mental health services for those below 12 years of age in Australia. In America, adolescent (also termed paediatric/child) mental health services can admit those from ages 5‐18 (Johnson et al., 2020; Kennard et al., 2018; Makki et al., 2018; Sams et al., 2016, 2018; Vidal et al., 2020; Wolff et al., 2018). Adult mental health services in Australia would typically be from age 18 onwards (Hu et al., 2019).

In a systematic review, Hayes et al. (2018) examined 16 studies of 30 national and international child and adolescent inpatient units to evaluate the effectiveness of care and found that the majority of adolescents improved in at least one area of symptomatology by discharge. The authors emphasized the need for further detail regarding adolescent inpatient unit operations and models of care (e.g., types of groups offered and description of experiences of adolescents participating in care). Features, which were listed included some demographic information such as age, gender, or the average length of stay of adolescents who were admitted. There was little to no information on therapeutic models of care, groups offered, and outcomes according to different therapeutic models nor the experiences of adolescents who engaged in those models. With limited evidence of effectiveness available, it is unlikely that high quality, safe, and effective inpatient programs simply occur or that any professional group could create a therapeutic milieu as well as any other (Delaney, 2017, 2019).

Adolescent inpatient literature tends to focus on symptomatology outcomes or rather than clinically and indeed in some instances non‐clinically, meaningful outcomes which are central to patient's experience of care (Steele, 2021). Therefore, while clinicians and researchers are informed of the clinical outcomes, details from how care starts, what is delivered within the care journey, and up to and including endpoints remain ambiguous; in turn, this limit how data are utilized. According to Fung et al. (2008), reporting of data is proposed as a mechanism for improving quality of care and safety by providing more transparency for clients and clinicians. Comprehensive and clear reporting is paramount for the translation of research findings into improved service delivery (Borek et al., 2015; Davidson et al., 2003).

To our knowledge, no comprehensive checklist has been developed to guide the descriptions and reporting of general adolescent mental health inpatient units. Research has supported the utility for such clinical practice checklists (Brouwers et al., 2016; Harris & Russ, 2021; Tokalić et al., 2020). For clinical practice guidelines, the international Appraisal of Guidelines, Research and Evaluation (AGREE) research team developed a checklist (Makarski & Brouwers, 2014). This has been widely used for evaluating methodological quality and transparency of practice guidelines internationally (Brouwers et al., 2016; Makarski & Brouwers, 2014). Clinical practice guidelines are systematically developed to assist clinician and patient decisions regarding appropriate health care for specific clinical circumstances (Brouwers et al., 2010). Furthermore, checklists can be, “used to translate evidence into practice by synthesizing strong evidence into actionable recommendations” (Francke et al., 2008; Sanders et al., 2014; Tokalić et al., 2020, p. 2167).

Shelmerdine et al. (2021) acknowledged that while reporting guidelines have been widely used, additional factors need to be evaluated which might not conform to traditional reporting guidelines. With changing health interventions, reporting guidelines can help ensure that investigators as well as those appraising studies can consider important features of good design and reporting (Shelmerdine et al., 2021). As we redesign health care systems in the context of the global pandemic and major mental health reforms across numerous nations, such a checklist may also be of benefit. Therefore, this paper presents a systematic review of the reported features of adolescent inpatient units to identify how these are defined and propose a preliminary checklist for reporting.

## 2 AIM

This review aimed to examine the operational and therapeutic model of care descriptions of general adolescent mental health inpatient units in the published literature and identify the key features which define them. The operational model relates to how and what is delivered as part of an inpatient unit. The model of care includes defining elements of the inpatient setting, which make up the model of care, including organizational structure, admission processes, and provision and delivery of all interventions. A secondary aim was to develop a preliminary checklist to utilize when researchers or clinicians report on an adolescent mental health inpatient unit.

## 3 METHODS/DESIGN

This review utilized the Population, Intervention, Comparison, and Outcome (PICO) framework to prepare the research question (Abbade et al., 2017). Electronic searches of CINAHL (Cumulative Index to Nursing and Allied Health), MEDLINE (Medical Journals), EMBASE (Medical Literature Database), ERIC (Education Resources Information Centre), and PsycINFO were undertaken. The search methodology and reported results adhered to the relevant sections of the Preferred Reporting Items for Systematic Reviews and Meta‐Analyses (PRISMA) statement (Moher et al., 2009).

### 3.1 Inclusion and exclusion criteria

The studies were included if the inpatient unit had a general focus, rather than for specialty areas such as substance abuse or eating disorder units. Adult and/or paediatric units were excluded. Studies were also included when the mean age of adolescent participants was between 12 and 25, studies were written in English and published between January 2000 and June 2021. The age range of 12–25 was selected as research has expanded the timeframe of adolescence to include young adulthood often up to 25 years of age (Jaworska & MacQueen, 2015). The previous 21 years were deemed sufficient based on the limited studies on adolescent inpatient units, and we also sought to include more contemporary inpatient units. Primary research of any design was included. Studies were excluded if the setting was based solely on community, outpatient, and/or forensic settings. Furthermore, studies which did not describe the inpatient unit or any therapeutic interventions, were excluded. The search was conducted twice, in April 2018 to resource an early version of this work and in June 2021 to review updated data with same search terms (May 2018–June 2021). On reviewer advice, one further paper was identified, which suggests this was associated with indexing. Full text screening showed that it contained no further fields for use in this analysis.

The following search terms were used for all five electronic databases: ([adolescen* OR “young person*” OR “youth*” OR “young adult*” OR teen* or child*] AND [inpatient* OR “in‐patient*” OR client* OR patient* OR “service user*”] AND [“mental health setting*” OR “inpatient unit*” OR in‐patient unit*“ OR hospital* OR admission* OR “mental health service*” OR “mental health*” OR “general” OR “general inpatient unit*”] AND [“Intervention*” OR “Therap*” OR “Treat*”]). Results were limited to peer‐reviewed journals where most reporting of this nature has occurred. The reference lists of included studies were hand‐searched to include all relevant empirical studies. When required, we contacted study authors to confirm eligibility and/or to acquire additional data.

We performed the initial search with identified terms and managed references with Endnote X9 software. (EndNote, 2019). We removed duplicate entries and sorted remaining articles. Article titles and abstracts were then double‐screened based on the inclusion and exclusion criteria, followed by full texts of selected articles. A randomly selected 20% subsample of full‐texts (*n* = 6) was double‐screened by (de‐identified for review). Following this screening process, one article was excluded. This process prompted further scrutiny of multiple site studies against the inclusion and exclusion criteria. No further studies were excluded.

### 3.2 Data extraction and quality assessment

Researcher (de‐identified for review) extracted data from full‐text papers using structured tables. These tables included details relating to characteristics of the study and inpatient unit, such as aim, research design, sample size, and sample characteristics (age, gender, ethnicity). The extracted data from all papers were assessed and screened for accuracy and any omitted information. Studies which did not describe characteristics of their inpatient units were excluded. To assess the quality of included studies, the relevant Critical Appraisals Skills Programme (CASP) tool was used (Critical Appraisal Skills Programme, 2018). The CASP tool considers the following issues when appraising a systematic review: are the results valid and what are the results are and will the results help locally.

### 3.3 Data analysis

A narrative review and synthesis approach was used to analyse the data (Popay et al., 2006). This method comprised four stages. These stages included developing a framework whereby criteria such as the aims and the type of intervention were identified, developing a preliminary synthesis of findings, exploring data relationships, and assessing the robustness of the synthesis (Popay et al., 2006). Narrative synthesis was chosen to “go beyond simple summation, map relationships in the extracted data both within and between studies…drawing upon different types of evidence” (Madden et al., 2018, p. 649). Furthermore, narrative synthesis methods can “bridge the divides between research, practice and policy” (Madden et al., 2018, p. 646). In the current review, this data analysis process was considered appropriate for the application of the findings to assist with bridging the adolescent inpatient gap between theory and practice.

## 4 RESULTS

### 4.1 Study selection

**FIGURE 1:**
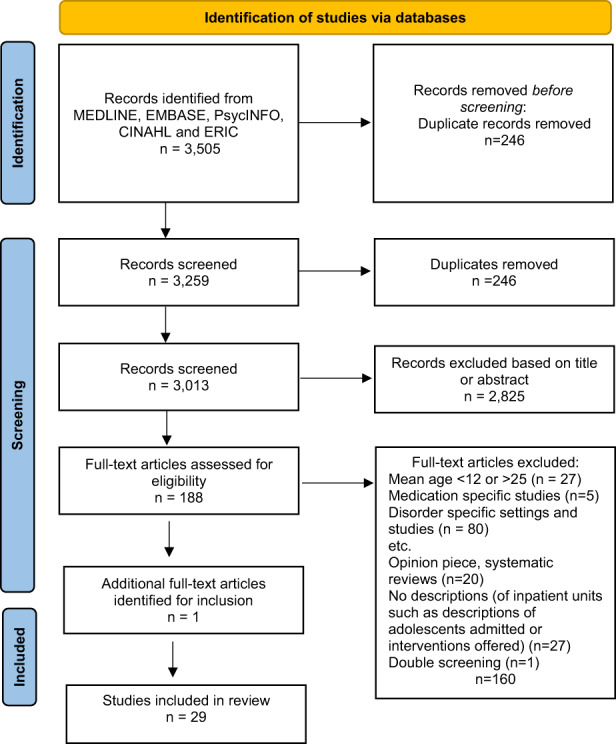
PRISMA flow diagram

The study selection process has been summarized in Figure 1. The search resulted in 3505 papers, whereby 492 duplicates were removed, resulting in 3013 papers. Of these, 2825 were removed as the titles and abstracts did not relate to the research aims. Of the 188 remaining papers, 28 met the inclusion criteria (see Figure 1). All studies were written in English and published between 2000 and 2021. The literature review was conducted in two stages, 2000–2018 and 2018–2021. One article, which we believe was ‘under review’ in the overlapping period in 2018 was later identified, met the inclusion criteria, and was subsequently included. A final search of the overlapping period was conducted to ensure no further studies were omitted. Details of included studies (*n* = 29) are provided in Table 1. Most of the studies were from the United States (*n* = 9) and Australia (*n* = 8), followed by New Zealand (NZ) (*n* = 4). Two studies did not provide their location. Fourteen studies were quantitative, eight retrospective chart reviews, five qualitative, and two mixed‐methods. Sample sizes varied greatly across studies, from two participants up to 1733.

**TABLE 1:** Summary of studies meeting inclusion criteria and sample description

Author (year), country	Aim	Design	Sample size	Age Mean	Gender (Female %)	Ethnicity (%)	Involuntary status (%)	Primary diagnosis (%)	Comorbidity (%)	Psychotropic medications (%)
Benarous et al. (2021) France	To examine length of stay and change in symptom severity and in the level of functioning	Retrospective chart review	106	M = 15 SD = 0.16	43%	Not available	Not available	100% mood disorders (only included in this study)	Mean 2.49 (SD 0.13)	Mean 1.81 (SD 0.93)
Bobier et al. (2009), New Zealand	Examines nursing and other multidisciplinary interventions commonly used	Quantitative	46 (consists of three diagnostic groups below) Mood (*n* = 22) Mixed (*n* = 15) Psychosis (*n* = 19)	M = 13.16 SD: Not available	Mood group: 64% Mixed group: 87% Psychosis group: 44%	Not available	Not available	Mood disorder: 48% Mixed disorder: 33%	Mood group: 64% Mixed Group: 60% Psychosis Group: 67%	Not available
Dean et al. (2007), Australia	Evaluate the impact of a milieu‐based behavioural management programme on the frequency of aggressive behaviours	Retrospective chart review	65: Pre‐intervention 86: Post‐intervention	Pre‐intervention M = 13.61 SD = 3.11 Post‐Intervention M = 13.59 SD = 4.28	Pre‐intervention 56.9% Post‐intervention 60.5%	Majority Not available Pre and post‐intervention 4.6% Aboriginal or Torres Strait Islander	Not available	Pre‐intervention anxiety disorders: 26.2% Post‐intervention unipolar depressive disorders and anxiety each: 19%.	Not available	Pre‐intervention: 66.1% (39/59) Post‐intervention: 65.4% (53/81)
Garner et al. (2008), Australia	Examine the effectiveness of a relaxation massage therapy programme in reducing stress, anxiety, and aggression	Quantitative	47 Treatment as Usual (TAU): 18 Massage Therapy (MT): 29	TAU M = 20.1 SD = 1.7. MT M = 20.9 SD = 2.3 TAU&MT M = 20.5	TAU 50% MT 44.4%	Not available	TAU 21.4% MT 61.1%	TAU Non‐affective Psychosis: 42.9% MT Borderline Personality Disorder: 27.8%	Not available	Not available
Guvenir et al. (2009), Turkey	Examine the treatment outcome in terms of patients functioning levels	Retrospective chart review	90	M = 15.3 SD = 1.9	67.8%	Not available	Not available	Mood disorders: 37.7%	Not available	100% Antipsychotics: 87.8% Antidepressants: 53.3% Mood Regulators: 22.2% Benzodiazepines: 42.2%
Hayes et al. (2020a), Australia	Explore adolescent and caregiver experiences of an inpatient model of care and perceived helpfulness	Qualitative	72	M = 16.2 SD = 1.61	81.9%	Caucasian: 91.7%	0%	Mood disorder: 56.9% followed by Anxiety disorders: 25%	73.6% (more than 1 diagnosis on discharge)	Not available.
Hayes et al. (2020b), Australia	To characterize adolescents admitted to voluntary adolescent inpatient units and investigate treatment outcomes	Retrospective chart review	72	M = 16.2 SD = 1.6	81.9%	Caucasian: 91.7%	0%	56.9% Major depressive disorder followed by anxiety disorders: 25%	75.7% more than one diagnosis on discharge.	Not available.
Hense et al. (2018), Australia	Use of a healthy–unhealthy use of musical scale as a single‐session music therapy intervention	Qualitative	23	Not available	Not available	Not available	Not available	Psychotic disorder: 56% Depressive disorder: 30% Bipolar disorder (13%)	Not available	Not available
Hottinen et al. (2020), Finland	Evaluate impact of the implementation of the safeward's model on the social climate on adolescent psychiatric wards	Quantitative	42: Pre‐implementation 39: Post implementation	Not available	Not available	Not available	Not available	Not available	Not available	Not available
Imran et al. (2021), Pakistan	Understand the various characteristics of children and youth admitted to inpatient unit and response to treatment	Retrospective chart review	634	M = 12.3 SD = 2.3	56%	Not available	Not available	Neurotic, stress‐related and somatoform disorders: 41.3% followed by Mood disorders: 12.3%	25% comorbid diagnosis of intellectual disability on Axis III	Not available
Johnson et al. (2020), United States	Evaluation of a substance use brief intervention for adolescents	Mixed methods	158	M = 15.81 SD = 1.24	54.4%	White: 67.1%, Other Race: 19.6%, Black: 13.3% and Hispanic/Latino: 22.@%	Not available	Not available	Not available	Not available
Kennard et al. (2018), United States	Pilot study of an inpatient intervention for suicidal adolescent to reduce suicide attempts following hospital discharge	Quantitative	66	M = 15.1 SD = 1.5	89.4%	Caucasian:77.3%	Not available	Major depression: 86.4%	57.6% with an anxiety disorder	Not available
Laget et al. (2006), Switzerland	Use of a multidimensional assessment tool in a psychiatric adolescent care unit	Quantitative	135 Inpatient Unit (IPU):68 General Population (GP): 67	IPU M = 15.5 GP M = 16.1 IPU and GP M = 15.8	IPU: 66% GP: 52.2%	Not available	Not available	Group 1: Behavioural & emotional disorders: 33.3% (*n* = 15) Group 2: Mood disorders: 36.8% (*n* = 19) Group 3: Mood disorders: 54.8%(*n* = 31)	Not available	Not available
Lee et al. (2018), United Kingdom	Outcomes of inpatient treatment	Quantitative	128	M = 16.1 SD = 1.33	71.1%	Not available	Not available	Not available	Not available	Not available
Makki et al. (2018), United States	Implementation of an ACT Curriculum on an Adolescent Inpatient Psychiatric Unit	Quantitative	T1 = 97 T2 = 83 T3 = 102	T1 = 15.47 T2 = 15.45 T3 = 15.76 T1,T2 & T3 = 15.56	T1 = 70% T2 = 77% T3 = 64%	Not available	Not available	Not available	Not available	Not available
McIlvain et al. (2015), United States	Piloting yoga and assessing outcomes	Quantitative	22	M = 15 SD = 1.47	64%	Not available	Not available	Bipolar disorder: 45.4%	Not available	Not available
Patterson et al. (2015), Australia	Assess feasibility of delivering a music therapy programme on adolescent psychiatric wards	Mixed methods	43	M = 15	25% of admissions are young women	Not available	Not available	Not available	Not available	Not available
Reynolds et al. (2016), Unknown	Examine the effectiveness of a modified version of the Positive Behavioural Interventions and Supports (M‐PBIS) implemented to reduce use of seclusion and restraint	Quantitative	726: Pre‐Intervention 759: Post‐Intervention	Pre‐Intervention M = 12.84 SD = 3.14 Post‐Intervention M = 13.53 SD = 2.66	55.2%	59.2% Black 31.8% White	Not available	Pre‐intervention: Depression: 31% (*n* = 225) Post‐intervention Depression: 35% (*n* = 262)	Not available	Pre‐intervention: 43% (*n* = 313) Post‐intervention: 31% (*n* = 234)
Saito et al. (2020), United States	Examine whether DBT significantly decreases depressive and manic symptoms compared with treatment as usual	Retrospective chart review	425: Dialectical Behaviour Therapy (DBT) Group 376: Treatment as Usual (TAU)	DBT Group M = 15.67 SD = 1.44 TAU Group M = 15.59 SD = 1.54 DBT & TAU M = 15.63	DBT Group: 66.3% TAU Group: 62.7%	DBT Group 40.9%: Caucasian 20.2%: Multiracial TAU Group 52.7%: Caucasian 22.1%: African American	Not available.	DBT Group Depressive disorder: 46.4% TAU Group Depressive disorder: 47.1%	Not available	Not available
Salamone‐Violi et al. (2015), Australia	Young person's perspective on inpatient unit	Qualitative	11	Not available	45%	Not available	36%	Not available	Not available	Not available
Sams et al. (2016), United States	Integration of a strength‐based approach with a traditional, medical model of psychiatric care	Quantitative	39	M = 15.46	76.9%	Not available	Not available	Depression: 61%	Not available	Not available
Sams et al. (2018), United States	Mindfulness‐based group therapy and impact on hospitalized adolescents	Quantitative	65	M = 15.06 SD = 1.34	73.8%	Not available	Not available	Depressive Disorder: 60% Mood Disorder: 21.5%	Not available	Not available
Spencer et al. (2019), Australia	Adolescents' experiences of distress on an acute mental health inpatient unit	Qualitative	7 (Adolescents)	Not available	85%	Not available	Not available	Not available	Not available	Not available
Swadi et al. (2010), New Zealand	Determine if patients undergoing treatment receive psychoeducation according to unit philosophy	Quantitative	60	Not available	Not available	Not available	Not available	Not available	Not available	Antidepressants: 61.7% (*n* = 37), Antipsychotic: 33.3% (*n* = 20) Mood Stabilizer: 15% (*n* = 9) Anxiolytic: 11.7% (*n* = 7) Hypnotic/sedative: 8.3% (*n* = 5)
Swadi and Bobier (2012), New Zealand	To determine the rate, indications, and process for using seclusion for patients undergoing treatment	Quantitative	50	M = 16.76	48%	56%: NZ European	Not available	Psychotic disorders:38%	Not available	Not available
Vidal et al. (2020), United States	To understand the risk factors for seclusion	Retrospective chart review	1733: No seclusion 253: Seclusion	M = 13.49 SD = 2.73	No seclusion: 19.8% Seclusion: 80.2%	No seclusion: 93% White Seclusion: 76% Black	Not available	Not available	Not available	Not available
Walker and Kelly (2011), Unknown	Introduction of an early warning signs journal	Qualitative	2	Not available	Not available	Not available	Not available	Not available	Not available	Not available
West et al. (2017), New Zealand	Evaluation of the use and efficacy of a sensory room	Retrospective chart review	112	M = 15.35 SD = 1.35	76.8%	Not available	Not available	Sensory room user: Depressive disorders: 71.4% Non‐sensory room users: Depressive disorders: 66.1%	Not available	Not available
Wolff et al. (2018), United States	Evaluate feasibility and efficacy of a psychosocial intervention.	Quantitative	463	M = 14.45 SD = 1.20	64.58%	74.34%: Caucasian 7.52% African American	Not available	Major depressive disorder: 69.55% Generalized anxiety disorder: 53.56%	M = 2.38 SD = 1.48	Not available

### 4.2 Quality of evidence

The CASP tool was used to assess the quality of the included studies (Critical Appraisal Skills Programme, 2018) (see Table 2). As this review included various study designs, the relevant CASP tool was applied. Ratings of “yes”, “no”, or “unknown” were used to indicate whether CASP items were demonstrated. The first author independently conducted the quality appraisal of included studies.

**TABLE 2:** Quality appraisal of included studies

CASP Checklist													
Author (year), country	Study Design	1	2	3	4	5	6	7	8	9	10	11	12
Benarous et al. (2021), France	Retrospective (Cohort)	✓	✓	✓	✓	✓	✓	N/A	N/A	✓	✓	✓	✓
Bobier et al. (2009), New Zealand	Quantitative (Cohort)	✓	✓	✓	✓	–	✓	N/A	N/A	✓	✓	✓	✓
Dean et al. (2007), Australia	Retrospective (Cohort)	✓	✓	✓	✓	–	✓	N/A	N/A	✓	✓	✓	✓
Garner et al. (2008), Australia	Quantitative (Cohort)	✓	✓	✓	✓	–	✓	N/A	N/A	✓	✓	✓	✓
Guvenir et al. (2009) Turkey	Retrospective (Cohort)	✓	✓	✓	✓	–	✓	N/A	N/A	✓	✓	✓	✓
Hayes et al. (2020a), Australia	Qualitative	✓	✓	✓	✓	✓	✗	✓	✓	✓	✓	N/A	N/A
Hayes et al. (2020b), Australia	Retrospective (Cohort)	✓	✓	✓	✓	✓	✓	N/A	N/A	✓	✓	✓	✓
Hense et al. (2018), Australia	Quantitative	✓	✓	✗	✗	–	✓	N/A	N/A	–	✓	–	–
Hottinen et al. (2020), Finland	Quantitative (Cohort)	✓	✓	✓	✓	–	✓	N/A	N/A	✓	✓	–	✓
Imran et al. (2021), Pakistan	Retrospective (Cohort)	✓	✓	✓	✓	–	✓	N/A	N/A	✓	✓	✓	✓
Johnson et al. (2020), United States	Mixed Methods (Cohort)	✓	✓	✓	✓	–	✓	N/A	N/A	✓	✓	–	✓
Kennard et al. (2018), United States	Quantitative (Cohort)	✓	✓	✓	✓	✓	✓	N/A	N/A	✓	✓	–	✓
Laget et al. (2006), Switzerland	Quantitative (Cohort)	✓	✓	✓	✓	–	✓	N/A	N/A	✓	✓	–	✓
Lee et al. (2018), United Kingdom	Quantitative (Cohort)	✗	✓	✓	✓	–	✓	N/A	N/A	✓	✓	–	✓
Makki et al. (2018), United States	Quantitative (Cohort)	✓	✓	✓	✓	–	✓	N/A	N/A	✓	✓	–	✓
McIlvain et al. (2015), United States	Quantitative (Cohort)	✓	✓	✓	✓	✓	✓	N/A	N/A	✓	✓	–	✓
Patterson et al. (2015) Australia	Mixed Methods (Cohort)	✓	✓	–	–	–	✓	N/A	N/A	✓	✓	–	✓
Reynolds et al. (2016) Unknown	Quantitative (Cohort)	✓	✓	–	–	✓	✓	N/A	N/A	✓	✓	–	✓
Saito et al. (2020), United States	Retrospective (Cohort)	✓	✓	✓	✓	✓	✓	N/A	N/A	✓	✓	✓	✓
Salamone–Violi et al. (2015) Australia	Qualitative	✓	✓	✓	✓	✓	✓	✓	✓	–	✓	N/A	N/A
Sams et al. (2016), United States	Quantitative (Cohort)	✓	✓	✓	✓	–	✓	N/A	N/A	✓	✓	–	✓
Sams et al. (2018), United States	Quantitative (Cohort)	✓	✓	✓	✓	–	✓	N/A	N/A	✓	✓	–	✓
Spencer et al. (2019), Australia	Qualitative	✓	✓	✓	✓	✓	✓	✓	✓	–	✓	N/A	N/A
Swadi et al. (2010) New Zealand	Quantitative (Cohort)	✓	✓	✗	✗	✗	✓	N/A	N/A	✓	✓	–	✓
Swadi and Bobier (2012), New Zealand	Quantitative (Cohort)	✓	✓	✗	✗	✗	✓	N/A	N/A	✓	✓	✓	✓
Vidal et al. (2020), United States	Retrospective (Cohort)	✓	✓	✗	✗	–	✓	N/A	N/A	✓	✓	✓	✓
Walker and Kelly (2011) Unknown	Qualitative	✗	–	–	–	–	–	✗	✗	–	–	N/A	N/A
West et al. (2017), New Zealand	Retrospective (Cohort)	✓	✓	✓	✓	✓	✓	N/A	N/A	✓	✓	✓	✓
Wolff et al. (2018) United States	Quantitative (Cohort)	✓	✓	✓	✓	✓	✓	N/A	N/A	✓	✓	✓	✓

*Note*: Number 1–12 relate to items of the CASP tool (dependent on the relevant studies). Yes (✓), No (✗), Unknown (–).

### 4.3 Adolescent samples

Twenty‐three studies reported on mean age. See Table 3 for study setting age ranges. For the *n* = 2 studies that included pre and post samples, the mean age was 13.2 and 13.5 years, respectively. For the *n* = 21 studies that did not, the mean age was 15.39 years. Remaining studies (*n* = 6) did not report mean ages. In terms of gender, 25/28 studies reported this. Of these studies (n = 25), 19/24 claimed that cisfemale accounted for more than 50% of the samples. In terms of ethnicity, nine/28 reported on this. For these studies (*n* = 9), samples were predominantly (>50%) (*n* = 7) Caucasian, and the remaining two were reported as Black (59.2%) and NZ European (56%). Details reported on the primary diagnosis, comorbidities, and psychotropic medications are presented in Table 1.

**TABLE 3:** Adolescent inpatient settings

Author (year), country	Single Site (S) or Multiple (M)	Capacity	Admission rates (Annually)	Setting age range	Clientele	Staff	Length of stay (M)	Client/Clinician ratios	Attached Services
Benarous et al. (2021) France	M (2)	15: Esquirol unit 15: Seguin unit	Not available	Esquirol unit (12–15 years) Seguin unit (15–18 years)	Adolescent mental health problems.	Not available	M = 100.66 days SD = 97.102	Not available	School
Bobier et al. (2009) New Zealand	S	8	Not available	16 to 18 years	Severe Psychiatric Disorder. Admissions Not Accepted: Conduct disorder or substance abuse.	Not available	Not available	Not available	Comprehensive, well ‐resourced service with day facilities, outpatients and inpatients.
Dean et al. (2007), Australia	S	10	Not available	Not available	Crisis presentations, planned diagnostic assessments, and brief intensive therapy.	Multidisciplinary team of medical, nursing, and allied health staff.	Not available	Not available	Not available
Garner et al. (2008), Australia	S	16	450	15–25 years	Range of diagnoses.	Four or five nursing staff are present on the unit at all times	M = 8.6	1:4	Not available
Guvenir et al. (2009) Turkey	S	10	Not available	Not available	Severe behavioural and emotional disturbances	Not available	Generally 8 to 10 weeks	Not available	The unit was also attached to CAMHS, which has an outpatient unit.
Hayes et al. (2020a), Australia	S	10–12	Not available	12–22 years	Range of mental health disorders and with capacity to participate in the programme	Multidisciplinary‐Registered Nurses, Endorsed Enrolled Nurses, Psychologist, Occupational Therapist and Psychiatrists	Range 1–67 days. Mean 28 (SD 15.82)	Not available.	Not available
Hayes et al. (2020b), Australia	S	10–12	Not available	12–22 years	Voluntary inpatient treatment.	Multidisciplinary	Range 1–67 days. Mean 28 days (SD 15.8)	Not available	Not available.
Hense et al. (2018), Australia	S	Not available	Not available	18–25 years	Psychosis, mania, depression, and suicidality	Not available	7–10 days	Not available	Not available
Hottinen et al. (2020), Finland	M (6)	Not available	2015 (423) 2016 (526)	13–17 years	Not available	Not available	Range 8–98 days	Not available	Not available
Imran et al. (2021), Pakistan	S	6	Not available	Not available	Not available	Psychiatrist, Clinical Psychologists, Play Therapist, Speech Therapist, Trained Nurses, and Other Support Staff	Mean 15.60 (SD = 6.3)	Not available	Not available
Johnson et al. (2020), United States	S	34	700 (non‐repeat)	12–18 years	74% admitted for suicidal ideation or behaviour.	Multidisciplinary team‐Psychiatrists, Psychologists, Social Workers, Nurses, and Milieu Staff.	Mean 9 days	Not available	Not available
Kennard et al. (2018), United States	M (2)	Not available	Not available	12–18 years	Recent suicidal ideation with a plan or intent. Recent suicide attempt.	Not available	Mean 2.1 weeks (SD = 2.3) Intervention & TAU group Mean 2.3 weeks (SD = 2.3) TAU Group	Not available	Not available
Laget et al. (2006), Switzerland	S	Not available	Not available	Not available	Suffering from serious mental health problems.	Not available	Not available	Not available	Not available
Lee et al. (2018), United Kingdom	S	12	Not available	13–18 years	Wide range of symptoms, histories, contexts, and needs.	Multidisciplinary team including psychiatrists, nurses, clinical psychologists, psychotherapists, occupational therapists, and social workers.	Range 1–609 days Mean 162.12 (SD = 136.48)	Not available	Not available
Makki et al. (2018), United States	S	15	Not available	4–17 years	Broad range of diagnosis including psychosis and autism, majority admitted for affective disorders and would benefit from education‐based approach. Deemed danger to self or others.	Not available	Total average: 9.2 days T1 = 9.2 T2 = 9.3 T3 = 8.5	Not available	Not available
McIlvain et al. (2015), United States	S	36	Not available	Not available	Not available	Not available	Not available	Not available	Not available
Patterson et al. (2015) Australia	S	12	230	14–17 years	25% of admissions are of young women with eating disorders, and psychotic, mood, and anxiety disorders are common.	Not available	12–14 days	Not available	As clinically appropriate, patients attend a hospital school and two structured group activities each days.
Reynolds et al. (2016) Unknown	S	12	420	Not available	Not available	Not available	M = 8.57 days	Not available	Not available
Saito et al. (2020), United States	S	Not available	Not available	12–17 years	Not available	Multidisciplinary treatment team. two attending Psychiatrists and psychiatry trainees (i.e., residents and child and adolescent fellows), two social workers, two psychiatric rehabilitation specialists, three nurses per shift and 3–4 mental health workers per shift.	DBT Group Mean = 8.36 days (SD = 8.09)	Not available	Not available
Salamone‐Violi et al. (2015) Australia	S	Not available	Not available	15–17 years	Psychiatric illness	Not available	4–15 days	Not available	Not available
Sams et al. (2016), United States	S	24	Not available	5–18 years	Not available	Multidisciplinary Team. Teachers are integral members of the team.	Not available	Not available	Not available
Sams et al. (2018), United States	S	24 beds	Not available	5–18 years	Not available	Multidisciplinary teams consists of psychiatrists, psychologists, multidisciplinary trainees (psychiatry, psychology, nursing, and social work), nurse practitioners, masters level social workers, registered nurses, milieu counsellors (“psychiatric technicians”, and activity therapists.	15.61 days (SD = 10.22, Range = 4–62 days)	Not available	Not available
Spencer et al. (2019), Australia	S	12	Not available	5–18 years	Not available	Multidisciplinary team comprising medical, allied health, and nurses routinely provide care.	Not available	2/3 adolescents to 1 nurse	Not available
Swadi et al. (2010) New Zealand	S	8	Not available	16–18 years	Admitted based on acuity of clinical symptoms, risk assessment, outpatient services, and carers' ability to cope.	Not available	Not available	Not available	Comprehensive and well‐resourced service with outpatient and day facilities and inpatient services
Swadi and Bobier (2012), New Zealand	S	8	Not available	16–18 years	Severe psychiatric disorder.	Not available	Not available	Not available	Comprehensive and well‐resourced service with outpatient and day facilities and inpatient services
Vidal et al. (2020), United States	S	12–15	2011–2015 (1986 admissions)	5–17 years	Acute treatment and stabilization‐voluntarily admitted by their parents	Not available	8 days *N* = 1986 (Mean 8.13. SD 5.462)	5 patients per nurse and psychiatric assistant.	Not available
Walker and Kelly (2011) Unknown	S	Not available	Not available	12–18 years	Severe mental illness. Need for assessment. Inability to manage in the community	Not available	Not available	Not available	Not available
West et al. (2017), New Zealand	S	20	Not available	12–18 years	Acute and chronic psychiatric problems	A Multidisciplinary Team provides assessment and treatment for adolescents.	Not available	Not available	Not available
Wolff et al. (2018), United States	M (2)	34	Not available	12–16 years	Imminent threat to themselves or others	Multidisciplinary Team. Milieu staff, Nurses, Psychiatrists, Psychologists, and Social Workers	M = 9.34 days SD = 8.79	Not available	Not available

### 4.4 Adolescent inpatient settings

Details of the inpatient unit settings are provided in Table 3. Twenty‐five studies presented single inpatient units (public and private), while the remaining four studies included multiple units. Twenty‐two studies reported capacity ranging from six to 36 inpatient beds. Five studies reported their annual admission rates which ranged from 230 to 700 people. Twenty‐three studies indicated the age range for their inpatient unit settings, which were between the ages of four and 25. For studies which identified the types of adolescents admitted (*n* = 21), terms commonly used were, ‘range of diagnoses,’ ‘severe psychiatric disorder,’ ‘crisis presentations,’ ‘voluntary inpatient treatment,’ ‘suicidal ideation,’ ‘acuity of clinical symptoms’ and ‘acute treatment’. Sixteen studies did not present details of staff working on the inpatient unit setting/s, and none reported on whether this included peer workforce members (for example, consumer and/or carer consultants or peer support workers). ‘Multidisciplinary Team’ was the most commonly used term, while ‘Teachers’ were included for those inpatient units with attached schools. One study identified the number of nurses per shift.

The average length of stay (LOS) was reported in 14 studies, with a mean LOS of 30 days. The LOS range was reported in six studies and ranged from one to 609 days. The client to clinician ratio was reported in three studies (1:3, 1:4, and 1:5). Some studies (*n* = 6) reported services such as outpatient services (*n* = 4) and schools (*n* = 2). Studies also reported on location of inpatient unit, referral sources, catchment areas, secure or non‐secure unit, unit aims, objectives, and philosophy. Of the 29 studies included in this review, some studies have described the same inpatient unit setting (Bobier et al., 2009; Hayes et al., 2020a, 2020b; Sams et al., 2016, 2018; Swadi et al., 2010; Swadi & Bobier, 2012). Of these, the same sample was used in two studies (Hayes et al., 2020a, 2020b).

### 4.5 Adolescent inpatient unit interventions

All studies described a range of interventions (see Table 4). ‘Therapy’ followed by ‘Family’ and ‘Individual’ were the most frequently used terms when describing interventions across all 29 studies. Three commonly identified intervention terms were ‘Behaviour Therapy’ (*n* = 9), ‘Pharmacological Treatment’ (*n* = 8) and ‘Group Therapy’ (*n* = 7). Finally, ‘Cognitive Behaviour Therapy’ (*n* = 4) and ‘Dialectical Behaviour Therapy’ (*n* = 3) were described intervention terms.

**TABLE 4:** Adolescent inpatient unit interventions

Author (year), country	Intervention
Benarous et al. (2021), France	Integrative approach for adolescent mental health problems is fostered with different types of interventions, including pharmacological treatment. Individual counselling, supportive groups, occupational therapy, and recreational and sporting activities. The indications of providing more structured interventions during the stay are discussed case to case (example Cognitive Behaviour Therapy, bodily meditation, psychodrama). Family therapy. Psychoeducation. A step‐down approach through the orientation to ambulatory daily care facility.
Bobier et al. (2009), New Zealand	Group, Individual and Family Interventions (Illness education, Anger Management, Stress Management, Relaxation, Medication Education, Lifeskills Acquisition, Alcohol and Drug Education, Communication Skills Training, Problem Solving Skills Training, Relationship Education, Self‐Awareness Education, Medication Education, Sport‐Related Activity, Art Related Activity, Adventure Activity, Group Outing, Individual Outing, Cognitive Behaviour Therapy, Behaviour Modification Programme, Individual Support, Family Counselling, Family Support Advocacy, Supportive Liaison/Advocacy, Community Reintegration)
Dean et al. (2007), Australia	Milieu‐based behavioural management program. Individualized patient management plans, early detection and prevention, staff training, reinforcement of appropriate behaviours, and intervention using the least restrictive option.
Garner et al. (2008), Australia	Behavioural approaches (example time out, distraction, limit setting and engaging with staff), PRN medication, and the use of seclusion and/or restraint where necessary.
Guvenir et al. (2009) Turkey	Treatment was multi‐model. Individual, parent, family, group, and psychological and physical therapy interventions, depending on the individual needs of the adolescent‐implemented by multidisciplinary team. The treatment work at the unit was linked with the context of therapeutic milieu, which refers to dyadic relationship of the patient with the treatment team and his/her peers.
Hayes et al. (2020 A), Australia	A range of evidence‐based group programs are offered from Cognitive Behaviour Therapy, Dialectical Behaviour Therapy to Mindfulness. The unit describes itself as DBT‐informed, while including other therapeutic interventions.
Hayes et al. (2020 B), Australia	A range of therapeutic interventions are provided with Dialectical Behaviour Therapy as the underlying theoretical basis of care. A more detailed description of the model of care is provided in article.
Hense et al. (2018), Australia	Healthy–Unhealthy Uses of Music Scale as a Single‐Session Music Therapy
Hottinen et al. (2020), Finland	The Safewards model suggests alternative methods for containment and ensures that the use of containment does not lead to further conflict. The model provides 10 interventions which include: Clear mutual expectations, soft words, talk down, positive words, bad news mitigation, know each other, mutual help meeting, calm down methods, and reassurance and discharge messages.
Imran et al. (2021), Pakistan	Comprehensive assessments in close collaborations with families and schools. Various treatment modalities including psychotropic medications, family therapy, individual therapy, parenting work, play therapy, and help in developing individualized education programme.
Johnson et al. (2020), United States	Daily comprehensive evaluations, milieu therapy, psychiatric medication management, daily group therapy, individual supportive therapy, family sessions, and case management.
Kennard et al. (2018), United States	As safe as possible (ASAP) intervention. Focuses on emotion regulation and safety planning. Four modules (chain analysis and safety planning; distress tolerance and emotion regulation; increasing positive affect through savouring and switching; and review of the skills, safety plan and app (BRITE). Three‐hour intervention delivered on unit. The BRITE app prompts adolescents to rate their level of emotional distress on a daily basis and provides personalized strategies for emotional regulation and safety planning.
Laget et al. (2006), Switzerland	The individual project encompasses therapeutic, educational, sport, and leisure activities. The global care considers physiological, educational, familial, and social aspects.
Lee et al. (2018), United Kingdom	Psychiatric assessment and medication, individual therapy from different therapeutic models and family therapy. All treatments tailored to the specific needs of each adolescent and family. Each young person has a case manager from the multidisciplinary team who coordinates their care and offers a supportive relationship throughout their admission. The unit runs a weekday programme that includes education, group therapies, and daily community meetings. Much attention is given to therapeutic milieu.
Makki et al. (2018), United States	An Acceptance and Commitment Therapy approach encourages individuals to practice compassionate self‐acceptance for uncontrollable conditions while taking committed actions toward creating the kind of life they most want to live. Interventions are designed to help individuals increase their awareness of environmental demands, their personal values, and to coexist with challenging thoughts and feelings while taking actions in the service of achieving necessary and desired life goals.
McIlvain et al. (2015), United States	Yoga was added to the programme twice weekly using the pre‐selected video. It was facilitated and monitored by a qualified recreation therapist, trained in the research protocol. All were expected to attend following physician medical clearance. As with every other programme, adolescents could refuse participation.
Patterson et al. (2015) Australia	Music therapy.
Reynolds et al. (2016) Unknown	Modified Positive Behavioural Interventions
Saito et al. (2020), United States	Dialectical Behaviour Therapy
Salamone‐Violi et al. (2015) Australia	Individual therapy, group therapy, family meetings.
Sams et al. (2016), United States	Multidisciplinary strength‐based approach to treatment. A structured treatment programme is designed to meet the social, emotional, psychological, and academic needs of patients. Programme elements designed to help patients learn distress tolerance and emotional regulation skills, distraction activities, relaxation and mindfulness techniques, social skills, and recovery strategies. Goals also include improved family communication with a focus on helping the family recognize strengths and validate the patient's feelings and experiences.
Sams et al. (2018), United States	A structured treatment programme is designed to meet the biological, social, emotional, psychological, and academic needs of patients. Programme elements carefully designed to help patients learn distress tolerance and emotion regulation skills, distraction activities, relaxation and mindfulness techniques, social skills, and recovery strategies. Goals also include improved family communication with a focus on helping the family recognize strengths and validate the patient's feelings and experiences. Mindfulness‐based group therapy. iMatter (Improve Mindful Attention Enhance Relaxation) includes several possible activities, such as mindfulness meditation, mindful movements (simple yoga poses), mindfulness activity (e.g. mindful eating and walking), breathing exercises, and closing activity.
Spencer et al. (2019), Australia	Nurses provided care and treatment to young people based around activities of daily living, worked alongside the allied health and education staff to run ward programs, and accompanied medical staff in clinical interviews with young people and their families. Genera; observations. Meal support therapy.
Swadi et al. (2010) New Zealand	Interventions Prior to Seclusion: PRN Medication, 1:1 Special Nursing, Room Boundaries/ “open seclusion” or high dependency unit, Low Stimulus Environment, Distraction/Physical Redirection and Calming/Verbal de‐escalation.
Swadi and Bobier (2012), New Zealand	Psychoeducation. Our philosophy and approach is to provide inpatients with timely information about various aspects of their illness in the hope that they will have better control and grasp of what happens after discharge.
Vidal et al. (2020), United States	The programme included group therapy and daily contact with physicians, a psychologist, occupational therapists, social workers and nurses. Positive Behaviour Reinforcement Systems.
Walker and Kelly (2011) Unknown	The treatment plan assists in maintaining a smooth and coordinated discharge and integration back into the community. Weekly multidisciplinary team meetings take place to discuss progress and future care planning.
West et al. (2017), New Zealand	The unit's sensory room contained a variety of sensory equipment including a rocking chair, weighted blankets, fidget toys, scented oils, candy and teas, pictograph cards (e.g. flashcards depicting pleasant or calming images), music, and projected images (e.g. bubbles floating or rivers running).
Wolff et al. (2018), United States	Coping, Problem solving, Enhancing life, and Safety planning (COPES)‐based on empirically supported CBT manual‐adapted for inpatient setting. Consists of four skill training modules.

### 4.6 Developing a checklist for reporting

The studies adopted a broad range of key features when describing inpatient units, which are portrayed in Table 5. Capacity (*n* = 22) and number of beds at each inpatient unit were the most prominent feature, as well as the type of clients *(n* = 22) being admitted for inpatient care. While focusing on clients, age range (*n* = 24) emerged as the second most commonly cited feature. Only six studies provided descriptions of attached services. Despite clients and capacity being key features, average length of stay was not often reported, as well as annual admission rates. Clinicians working on the inpatient unit received little or no attention in 19 studies, with even less focus on clinician/client ratios (*n* = 3). There was a complete absence of mention of peer workforce support models.

**TABLE 5:** Key features and checklist for reporting on adolescent mental health inpatient units

Checklist for Reporting on Adolescent Inpatient Units (CRAIU)					
Item	Yes	No	N/A	Information Not available	Response and/or Remarks
Country and location of inpatient unit					
Catchment area					
Admission rates (Annually/Last calendar year)					
Readmission rates					
Unit capacity					
Referral source/s					
Length of stay (Average)					
Age range					
Gender Same sex Mixed					
Predominant gender					
Admission criteria Outpatient assessments (less than 1 month) for programme suitability prior admission? Were goals and expectations set with adolescents' prior admission? Inclusion and exclusion criteria (Who would be excluded and why?)					
Predominant diagnosis					
Secure status of unit, For example, High Medium Low Open					
Open or closed groups					
Staffing and equivalent full time Clinically trained staff Non‐clinical care staff Peer workforce					
Adolescent/Staff ratios Adolescent/Registered Nurse Morning shifts Afternoon shifts Night shifts Adolescent/Allied health					
Outcome measures routinely used (repeated)					
Intervention/s provided For example, Individual Group Milieu Family services Parent groups Sibling groups Is participation mandatory?					
Staff delivering interventions How often?					
Attached services For example, Medical School Outpatient Allied health services Family services Family accommodation					
Facilities For example, Onsite gym Sports hall Kitchen Sensory room De‐escalation room					
Discharge Procedures For example, Follow‐up appointments Ongoing therapy/therapies Planned readmission					
Treatment framework or model of care For example, Trauma informed care Cultural safety Patient‐centred					
Unit aims, objectives and stated mission					
Unit philosophy					
Funding source For example, Private State funded Private and state funded					
**Total**					

## 5 DISCUSSION

### 5.1 Main findings

We have systematically identified and appraised studies of general adolescent mental health inpatient units. The results indicate that general adolescent mental health inpatient unit studies do not provide comprehensive and consistent information to inform readers of their unique operations and models of care, nor the extent to which these influence effectiveness and outcomes. Young people are at the core of adolescent inpatient care and mental health, and broader health service policies and practice implore their inclusion in service design and treatment decision‐making to support more than engagement (Bjønness et al., 2020; Dent & Pahor, 2015; Storm & Edwards, 2013; Tindall et al., 2019; Viksveen et al., 2017). It is unclear from the studies reviewed whether adolescents were involved in making decisions about their own care. Current descriptions of inpatient units fail to demonstrate this. For instance, admission criteria, such as those ‘accepted’ or ‘excluded’, is an important factor to consider when capturing the acuity of a sample. This information could influence what mental health clinicians are considering in terms of therapeutic and supportive program implementation and suitability. Adolescent inpatient units should tailor services to the types of adolescent being admitted to maximize potential engagement (Brownlee et al., 2017). They should also allow for meaningful input into the models of care that are offered.

The ‘admission criteria’ feature can help gauge the different organizational pressures that each unit experiences and reflect how they function. It can be influenced by factors such as whether the unit is publicly or privately funded. For example, a public inpatient unit might be generally crisis‐driven, with consequent pressures related to crisis response and discharge to community services as soon as possible with little room for actual ‘therapy’. These ‘outside’ influences based on public/private, crisis, or therapeutic admissions all play a key role in describing adolescent inpatient units and should be considered when reviewing adolescent inpatient studies.

Similarly, identifying the primary diagnosis of adolescents being admitted can influence program planning. For instance, if adolescents admitted were predominantly diagnosed with Borderline Personality Disorder, DBT might serve as a significant component of the inpatient program (McCauley et al., 2018; Mehlum et al., 2014; A. L. Miller, 2007). Therefore, more detailed descriptions of the adolescent sample would be useful when reviewing adolescent inpatient unit studies with a greater emphasis on those experiencing it. In turn, this can inform how services are improved and settings redesigned in the future with the current emphasis in some countries, particularly Australia, on mental health service reform (Department of Health, 2021; State of Victoria, 2021).

Capacity of the adolescent inpatient unit is another important descriptor. Several adolescent studies have emphasized the value of peer support (Hayes et al., 2020a; Hayes, Simmons, Palmer, et al., 2019; Hottinen et al., 2020; Salamone‐Violi et al., 2015). Information related to the inpatient unit capacity and group therapy size could be useful for interpretation and what could be considered the ‘ideal’ unit or group size. Other descriptors include ‘referral sources’ and ‘location’. Location of the inpatient unit can indicate the socioeconomic status of adolescents being admitted. This is important for assessing the relationship between a particular inpatient unit and the types of adolescents being treated. Unfortunately, little research exists on the potential impact of socioeconomic status on readmission, despite correlations between low socioeconomic status and risk for first‐time admission (Kalseth et al., 2016; McLaughlin et al., 2012; Miller et al., 2020). However, it is likely that an inpatient unit in a higher socioeconomic status area will have more available resources and attached services than those in lower socioeconomic status areas.

To understand an inpatient unit, identifying the ‘Attached Services’ or ‘Facilities’ is important when considering the unit's available resources. This has been emphasized in the wider literature, as understanding the available resources plays a key role in implementing Evidence‐Based Practice (Alatawi et al., 2020; Bach‐Mortensen et al., 2018). Similarly, the inpatient unit's average ‘length of stay’ or ‘Repeat Admissions’ are important features, which need to be considered when evaluating an inpatient unit. Furthermore, other features such as trauma‐informed and culturally safe practices and policies. These features are important for supporting recovery focused practice across mental health care settings (Hawsawi et al., 2021). The absence of these key features creates many barriers for readers when attempting to compare and apply findings to their own inpatient unit settings.

Mental health clinicians and researchers should consider attached services and average length of stay for program considerations. For instance, what programs can be delivered within a certain time‐frame, based on the average length of stay. In a recent study, Larsen et al. (2020) aimed to create a flexible outpatient mental health transitions program. The study found that the attached outpatient service resulted in lower inpatient length of stay, reduction in readmissions, and improved therapeutic outcomes for patients (Larsen et al., 2020). Given the potential influence of these attached services, it is important that inpatient units are not considered in isolation, but part of a system of care. Considering the system of care, other pre‐admission descriptors would be beneficial such as information regarding pre‐admission assessment of adolescents, experience, and duration of previous treatment. This can aid the assignment of more appropriate interventions and individualized treatment during an inpatient stay.

Child and Adolescent Mental Health (CAMH) nurses constitute the primary workforce of inpatient units. There is no single definition of the CAMH role (Rasmussen et al., 2012). Despite the absence of any definition, CAMH nurses play a pivotal role in the inpatient experience and the therapeutic relationships they form with young people. Nurses are available 24/7 to support adolescents when experiencing any distress (Hayes et al., 2020a). Equally, other members of the interdisciplinary team facilitate and deliver the inpatient program, including consumer consultants in some models and peer support from the hospital generally to the inpatient unit. Despite their valuable roles on inpatient units, several studies exclude them in their descriptions. To understand an inpatient unit, information regarding staff (clinical and non‐clinical) working within the service would be of benefit. This could include the number of consultants admitting to each inpatient unit as well as the availability of a psychiatric registrar; adolescent to nurse ratios; and details of training, provided to clinicians.

In terms of interventions provided on each inpatient unit, there appeared to be much overlap in the descriptions. Similar terms were often used, such as ‘group’ or ‘individual therapy’. While these interventions are undoubtedly important, few mentioned (*n* = 6) whether the inpatient unit was based on a particular theoretical model, such as the principles of DBT or CBT. In the absence of further information, similar to the role of CAMH nurses, the interventions on adolescent inpatient units might be difficult to measure. Features such as ‘Unit Aims and Objectives’ and ‘Unit Philosophy’ might help differentiate the different units, helping create their own unique identity.

Evaluation literature is relatively rich on discussions of best practices in methods (Sridharan & Nakaima, 2011). However, Sridharan and Nakaima (2011) recommend a similar dialogue on what constitutes adequate program theory that can be implemented. Existing studies in adolescent inpatient literature often have sparse descriptions, making it difficult for others to replicate a study or emulate the methods in a new study. For this reason, we sought to assist the descriptions of adolescent inpatient unit studies. The insufficient information regarding each inpatient unit translated to a useful preliminary checklist. We suggest that the proposed checklist could be utilized when describing and reporting general adolescent inpatient units. Furthermore, the checklist can be utilized by key mental health stakeholders to prepare for the re‐design and improvements or the evaluation of an inpatient unit. The preliminary checklist is brief which is also a strength. It provides a framework for evaluation and can be adapted to context‐specific factors and therefore be broadly applicable. The checklist can also be employed by researchers and clinicians, so its application is flexible and, as a synthesis of available information, it is more detailed than existing resources.

The evidence available in Tables 1 and 3 demonstrate ambiguous adolescent inpatient services, which is potentially frustrating for readers. The resultant inability to utilize information contained in the articles is limited, which would not be the author's intentions. From a clinician and researcher perspective, when reading these articles, there is an attempt to understand the unit from the experience of the adolescent. There is an interest in not only the positive features but the negative features to be avoided.

Adolescent inpatient clinicians and researchers are likely to need this content to understand the service, compare it with theirs, and find the data clinically useful, thus enhancing relevance of such studies. Hence, the preliminary checklist offers further opportunities to share clinically relevant information with other adolescent inpatient services. Answering some of these key questions offers an opportunity for the reader to clearly understand a service and adopt suitable ideas from these studies. While research serves many purposes, clinical research needs to meet the needs of those at the forefront, adolescents and clinicians themselves.

The features listed in Table 5 were extracted from each study when describing their inpatient unit settings. The features used to define adolescent inpatient units in Table 5 present a useful checklist for individuals who wish to report on adolescent inpatient units. The checklist poses many questions in what features are considered the most important for those reporting on inpatient units. Limitations of how some studies report are clearly visible, leading to complications in terms of interpretation and evaluation. For example, a study reporting positive therapeutic outcomes for adolescent inpatients might present limited descriptions of their inpatient unit. This makes interpretation challenging for nurse unit managers and clinicians attempting to establish whether such findings can translate to their own inpatient setting. The absence of information limits the application of theory to practice.

The preliminary checklist aims to encourage mental health inpatient unit staff and researchers to consider what features they believe are important when capturing inpatient units. This checklist can provide a guide for reporting adolescent inpatient units, ultimately improving consistency and clarity among studies. The checklist was reviewed by a number of national and international adolescent inpatient experts. There were five national and international adolescent inpatient experts, which represented both clinical and research experience. Expert feedback was sought to ensure important universal features were listed to maximize potential use in various countries. An example of feedback to inform the checklist was one expert who believed the following information was important for the checklist:

- Percentage of patients under mental health act.
- Admissions planned/elective and/or acute.
- Admission criteria.
- Diagnostic groups treated.
- Details of professional groupings of clinical staff.

The checklist could be used as a flexible prompt for those reporting on adolescent inpatient units. In particular, the checklist can be applied to government policy writers for consideration of reform and quality improvement efforts. The checklists could be useful for journal editors to recommend reporting expectations of submitted manuscripts.

### 5.2 Limitations and strengths

The eligibility criteria excluded articles not written in English; thus, descriptions of adolescent inpatient units for other cultures were excluded. Studies included all have methodological limitations; however, the purpose of this review was to assess how general adolescent inpatient units are described. Disorder‐specific settings were excluded, which might have removed promising descriptions of alternative settings, such as eating disorder inpatient units, which may have had information, which coincides with general adolescent inpatient units. The content of the proposed checklist is limited by the content of the existing studies from which it was compiled, which in turn, is influenced by limited evidence. Nonetheless, the checklist could still be considered broad, and perhaps applied to other settings, such as adult inpatient units.

International experts reviewed the preliminary checklist for relevance and offered feedback for the checklist. The main strength of the review is that, to our knowledge, this is the first time a preliminary checklist has been developed to improve descriptions of general adolescent mental health inpatient units. Further research is required to expand this checklist for specific adolescent inpatient unit settings. In addition, the checklist has not been validated in any particular inpatient unit setting. Consequently, further validation is invited to improve evaluations of adolescent inpatient units. Many adolescent inpatient units care for adolescents up to the age of 25. However, the emerging trend toward a focus on the 12–25 or 15–25 age range means that the degree to which the checklist is useful for these services is limited until more data are available (Colizzi et al., 2020; O'Keeffe et al., 2015).

### 5.3 Implications

This review informs mental health nursing research by demonstrating the complexity of adolescent inpatient care and its unique features. The checklist can be used to improve reporting and encourage future research into this area (e.g. validation of checklist). For clinical purposes, unit managers and other senior staff can review existing services and plan for new ones using the checklist to consider how the service might function (e.g. discharge procedures) and what might be offered within it (e.g. including facilities such as a gymnasium or kitchen).

The checklist can help policymakers to make informed decisions about service planning and implementation (e.g. admission criteria). Although there are limitations in that the checklist has not been validated, we hope that it will inspire future efforts to critically appraise the ways in which research in adolescent inpatient settings is conducted and reported. This checklist was developed in response to the lack of information provided on research in adolescent inpatient units. Therefore, we hope to see an improvement in the level of information provided in reports on studies conducted in adolescent inpatient units. This will, in turn, assist with quality improvement, through a more detailed description of each setting, in addition to the nomination of areas that require further evaluation. The checklist can promote quality, safety, and greater focus on patient experiences during an admission. This includes increased discussions among clinicians regarding the mission, vision, and values of the organization in an effort to enhance the delivery of their inpatient service.

## 6 CONCLUSION

The current review has identified that adolescent inpatient units are poorly described, providing insufficient information to inform best practice. Consequently, we have developed a preliminary checklist to improve study design, execution, and generalisability of results. Further development and rigorous validation of the checklist is required to elaborate on all components and demonstrate relevance to a wider range of researchers and clinicians. Nonetheless, the preliminary checklist can result in improved planned evaluations of inpatient units and assist in developing more rigorous evaluation and implications for practice. Similarly, peer reviewers and editors can use the checklist as guidance to gauge the completeness and transparency of a systematic review protocol submitted for publication in a journal or other medium. The work presented in this review should be considered the first step in a longer‐term process of subsequent research. The main strength of the review is that, to our knowledge, this is the first time a preliminary checklist has been developed to improve descriptions of general adolescent mental health inpatient units.

## 7 RELEVANCE STATEMENT

This systematic review addresses a relevant and understudied issue for mental health nursing by addressing an important gap in adolescent inpatient literature. This aims to bridge the gap between theory and practice in relation to reporting of adolescent inpatient units. Based on the results, we propose the preliminary checklist for reporting of general adolescent inpatient units to improve clarity and consistency in reporting of adolescent inpatient services.

## AUTHOR CONTRIBUTIONS

All authors contributed to the design of the research, to the analysis of the results, and to the writing of the manuscript.

## CONFLICT OF INTEREST

We, the authors declare that there is no conflict of interest or ethical issues in the production of this descriptive literature review.

## ETHICAL APPROVAL

This research was partially funded by Ramsay Healthcare. However, the funders were not involved in the decision to publish or preparation of the manuscript.

## Data Availability

The data that support the findings of this study are available from the corresponding author upon reasonable request.
